# The New York Institute of Stomatology

**Published:** 1901-04

**Authors:** 

**Affiliations:** The New York Institute of Stomatology


### THE NEW YORK INSTITUTE OF STOMATOLOGY.

A REGULAR meeting of the Institute was held at the office of Dr. R. H. M. Dawbarn, No. 105 West Seventy-fourth Street, New York, on January 4, 1901, the Vice-President, Dr. A. H. Brockway, in the chair.

The minutes of the previous meeting were read and approved.

### COMMUNICATIONS ON THEORY AND PRACTICE.

*The Chairman.*—Doubtless most of us have occasion sometimes to remove amalgam fillings, and this is usually regarded as a difficult and tedious operation. I have been asked to describe a method which I have employed for many years, and which has proved very simple and efficacious. It consists simply in holding a heated instrument on the filling until the heat is felt in the tooth and then at once burring it out before it again becomes cold. It can be cut like cheese. I use for the purpose an instrument having a very slender shank with a bulbous end the size of a pea,—one of the old Woods metal fillers of forty years ago. This is admirably adapted for the purpose, as the slender shank prevents the heat from radiating too rapidly.

*Dr. J. Morgan Howe.*—Can this method be employed with all kinds of amalgam fillings?

*Dr. Brockway.*—Possibly palladium or copper amalgam might prove a little refractory, but I am not sure.

*Dr. F. Milton Smith.*—Since Dr. Locherty, of this Institute, read his paper on the removal of pulps by the use of cocaine and pressure, I have been somewhat relieved in my practice. Dr. Locherty was not the first to suggest the use of cocaine, but he was the first to impress it upon my mind as a matter of practice, and so to Dr. Locherty belongs very much of the credit for the comfort which I am now getting from this method. I am one of the unfortunates who have used arsenic for devitalizing teeth. I understand some men have never used it; but unfortunately several years ago I formed the habit, and somehow I seemed to be tied to it. Quite a number of years ago our esteemed friend, Dr. Gillett, showed us a method, in a clinic, for removing the pulp of teeth with cocaine, using the hypodermic syringe. I was not so successful with that method as I have been with the use of the crystals of cocaine forced into the pulp-cavity with pressure. Three or four days ago a lady came to me with an exposed pulp in a superior canine. She was in an extremely nervous condition, and it seemed as if she were going to jump out of the chair when I even attempted to dry out the cavity with bibulous paper. After gaining her confidence and adjusting the rubber dam, and after removing the loose *débris* in the cavity and drying it out, I applied crystals of cocaine moistened with carbolic acid. Then, having filled the palatal portion of the cavity with impression-material, I brought pressure upon the pulp by means of a pellet of softened gutta-percha. At first the patient was inclined to jump, but I insisted that she must keep still for a second, when she would have no further occasion to complain. In a few moments I was enabled to remove the pulp absolutely without pain. I speak of this simply to impress upon you that this is not an experiment, but a method of practice. We are too apt when we hear of new methods of this kind to let them go into one ear and out of the other. I think it is three months since Dr. Locherty read his paper before the Institute, and since that time I have used cocaine exclusively and have not used arsenic once for the devitalization of teeth. I wish thus publicly to thank Dr. Locherty for impressing upon me this valuable suggestion.

*Dr. H. W. Gillett.*—I want to supplement Dr. Brockway's suggestion. The large gutta-percha instrument which comes with the Russell electrical appliances will be found serviceable for the same purpose. I also find it very useful in testing pulp conditions.

Dr. Smith referred to me in connection with the removal of pulps with cocaine. First, I wish to enter a disclaimer as to its being an original method with me. My knowledge of it came through Dr. E. C. Briggs, of Boston. I wish to correct a misapprehension which Dr. Smith seems to be laboring under in regard to the method employed by Dr. Briggs and myself. The hypodermic syringe in this operation is not used in the same manner as in a hypodermic injection. The method differs from that described by Dr. Locherty only in that the pressure is produced by means of the piston of the syringe instead of a pellet of gutta-percha. There is no mechanical crushing of the pulp such as would result if a needle were passed directly into it. Of late I have been using in a large proportion of cases the same process that Dr. Smith mentions. I feel that it is a most objectionable practice to take a pulp from an anterior tooth by any other means, owing to the better results as regards discoloration.

*Dr. Charles O. Kimball.*—I was showing one of our members, our honored president, in my office the other day, an instrument which I have used for many years. It interested him, and so I have brought it here to show you. It is not in the market. I made it myself and have used it for thirty years. It is simply a stick-holder, but its superiority lies in its simplicity, the firmness with which the stick is held, and its ease of adjustment. The instrument is used in the cleansing of teeth. After the use of the little disks, brushes, etc., there are always little points which we cannot get at, and it is here that the convenience of this instrument is appreciated. I showed it to "Whites" years ago, but they did not take it up. Grafrath has been making them for my associates for some years.

*Dr. C. D. Cook.*—Codman & Shurtleff, of Boston, long ago made an instrument similar to this. The piece of wood is held in place by means of a claw clamp, the shaft of which runs through a small handle and is tightened by means of a screw or nut at the end. I have used one of them many years.

*Dr. Kimball.*—The great advantage of this instrument is that there is no screw, and consequently the readjustment can be effected by one hand. The instrument being held in the middle of the hand, the knob at the end can be moved with the thumb and finger.

*Dr. J. Morgan Howe.*—I was quite interested in the simplicity of the instrument when Dr. Kimball showed it to me, and I would like to ask Dr. Kimball if he would kindly have a drawing made for an illustration, so that the instrument may be copied.

*Dr. S. E. Davenport.*—It has recently come to the attention of the Executive Committee that there are a number of Institute members who are not subscribers to the INTERNATIONAL DENTAL JOURNAL. Standing as we do for independent journalism, it hardly seems loyal to our principles not to subscribe to practically the only independent dental journal in existence. Inasmuch as in this journal four very important dental societies report their proceedings,—viz., The Harvard Odontological Society and the American Academy of Dental Science, of Boston, the Academy of Stomatology, of Philadelphia, and our own society, it seems to me that those who do not subscribe are not only lacking in loyalty, but are losing a great deal of valuable reading matter. The members of the Executive Committee hope that all members who are not subscribers will become so at once, if for no other reason than to enable them to read the proceedings of their own society when they are obliged to be absent from its meetings.

*Dr. Howe.*—It does not seem necessary for me to emphasize anything which Dr. Davenport has said, but I wish to add any weight I may be able as to the importance of our support of the INTERNATIONAL DENTAL JOURNAL. The societies that publish their reports in the JOURNAL are certainly expected to support it, and I feel that every one here who is not a subscriber should feel it his duty to subscribe at once.

As Dr. Bogue, who was to have given us a talk upon the subject of the International Dental Congress, at Paris, is confined to his bed, he has been kind enough to write for us most of the matter he intended to present, and the secretary will read it for us.

(For Dr. Bogue's paper, see page 218.)

*Dr. G. F. Eames.*—Experiments and observations on the varying conditions of the saliva and other oral secretions should be of great interest to the dental profession, for I believe that a better understanding of these conditions will give us the key to the proper treatment of many pathological conditions which are the result, not the cause, of a change in the character of the oral secretions. It is my belief that disturbances of the oral fluids may be caused by a local irritant, but I also believe that they more commonly have a constitutional origin. We shall all agree, of course, that a local cause, which produces a local disturbance, may in various ways be communicated to the general system and become constitutional. I regret that I am unable at the present time to speak at length upon this important subject. I can only wish each of you a Happy New Year and much prosperity as a society.

*Dr. Gillett.*—In regard to the last item mentioned by Dr. Bogue, that of a single support for a bridge, I have been employing that principle for some time past with great satisfaction. The device which I have been using has been described in the dental journals as the Stowell bridge. The idea of a bridge with only one fixed cemented attachment is a most admirable suggestion.

*Dr. Platt.*—I am very much interested, as we all are, in this line of practice, and while I do not feel competent to speak authoritatively on the subject, I would like some information as to method. I have for some time past felt that we need a change in our mode of practice. I have had good success with short bridges carrying one or two teeth, with one firm attachment and a lug. I have had one or two failures where the strain has been so great that they have pressed into the gum. I have found that with comparatively long bridges, with two or more attachments, they almost invariably would break loose from all but one of the attachments, and how to overcome this has annoyed me very much.

*Dr. J. B. Locherty.*—I have done some bridge-work in the past few years, but so far as the "breaking away" of a bridge is concerned, it seems to me that if the roots are strong enough a bridge can be properly made and adjusted which will be firm and satisfactory in every respect. Dr. Gillett's method I should like to know more about. I have been practising those which have been in vogue for a number of years, but of course in this work we cannot follow any one method; we have to use our own judgment. I have made bridges extending from the cuspid to the second or third molar, placing a gold crown on the molar and a so-called Richmond on the cuspid. I recall one such bridge in the mouth of a patient, a very large man, which made it necessary to make a correspondingly strong bridge. It has been in use some years now, and is in perfect condition. With regard to the apparent sacrificing of teeth, I never hesitate to do so; that is, I never hesitate to remove the pulp and crown of a tooth where I think a permanent and satisfactory bridge can be applied; for I contend that if a pulp is removed and a crown is nicely fitted upon the root, the removal of said pulp will in no way shorten the life of the root, for in many instances a tooth will have a tendency to tighten and become firm after the extraction of a pulp where previously through various causes it was not firm. In adjusting a gold crown or a porcelain crown of the Richmond style a very satisfactory method is to first paint the crown with chloro-percha, then, if the bridge has ever to be removed, it is not so difficult. It seems to me that we have to sacrifice something in order to gain something, and I think the sacrifice is justifiable in many instances.

*Dr. G. E. Rice.*—Two cases came into my hands during the past year of a similar nature, both bridges with a crown on a molar as one anchorage, a poor first bicuspid root fitted with a band and pin as the other, and a history of continued annoyance on account of the breaking loose of the anterior attachment. In the first case I fitted a pin to the bicuspid root, covered the end of the root with platinum, soldered pin and platinum base together, and then built up a knob of gold plate and solder. This was cemented in place with oxyphosphate of zinc, and presented much the same appearance as the round top of a brass-headed picture-nail. The under side of the anterior end of the bridge was cupped to fit on this metal surface, forming a half ball-and-socket joint. This bridge has been in place about ten months, and is doing good service. The second case was treated in a similar way five months ago, and is in good condition. It would appear that this method is worthy of consideration for short bridges where a small weak bicuspid root has to be used as one anchorage.

*Dr. H. W. Northrup.*—In my early experience with bridge-work I used to have a great many break-downs, and gradually learned that my failures were due to a lack of sufficient amount of material to make the work strong. Of course, in this line of work we are not able to judge the amount of strain which the jaw will exert. This difficulty has been overcome entirely by using a sufficient quantity of material, especially solder. I think the heaviest bridges that I ever made were for a physician in this city. They were a full upper and lower bridge, carrying fourteen teeth each. On these two bridges I used thirty-seven pennyweights of solder, besides two or three pennyweights in making the caps and root attachment. The bridges have been in the mouth five years and are giving perfect satisfaction.

*Dr. Gillett.*—A great deal of our discussion has been along the line of the breaking of the bridge itself. Of course the breaking of a bridge is very troublesome, but it is not this particular point which interests me most. I find such breakage easy enough to prevent, but a rigid bridge often results in loosening of the abutments and irreparable damage. I firmly believe that the principle of one fixed point of attachment, with sufficient support at the other end to withstand the strain of mastication, is going to play an important part in our future work.

*Dr. Howe.*—We have had a very interesting discussion on this subject of bridges, and also upon the subject of the analysis of the saliva. The latter especially is a very important subject, and I hope Dr. Michaels's experiments will be pursued by others.

*Dr. F. Milton Smith.*—May I correct what possibly might have seemed to be a reflection on Dr. Gillett's method as shown to us years ago? Whenever I speak extemporaneously I am apt to put my foot in it. I certainly misunderstood Dr. Gillett's plan, as demonstrated then. I should judge from his suggestions that it is practically the same as the method which I use. I therefore wish to apologize to Dr. Gillett. I have learned so much that is valuable from him that I am the more willing to do so, and also to thank him for the other things which I have learned from him. But regarding bridge-work loosening at one or the other end within a very short time, I must beg to differ with Dr. Gillett. I do not believe this is the case where the bridge is properly applied. If so, why is it that we see so many cases of bridge-work lasting from two to twenty years and with three and even four attachments. There is no question that they do loosen sometimes, this all of us are willing to admit. Is not the trouble here, that we often ask too much of two or three weak teeth? Certainly in most things there are bounds beyond which we cannot go. Another point, which Dr. Northrup has also mentioned, is the fact that often the work is not strong enough. I cannot understand how a person can expect good work from a bridge which has not sufficient strength.

*Dr. Northrup.*—I would like to mention one case which came to my notice recently which would tend to prove that bridges do not loosen the teeth, but that they are beneficial to them. It was that of a patient with molar, canine, and central on one side of the upper jaw, and a central, lateral, and canine on the other. A bridge connected this molar, canine, and central. All these teeth were affected with pyorrhœa. I had the case under observation for about three years, and noticed that the teeth which supported the bridge were much stronger and firmer than the others and lasted some weeks longer.

*Dr. C. F. Allan.*—Some months ago I had occasion to remove a bridge of somewhat peculiar construction from the mouth of a patient. This bridge carried only one tooth, a bicuspid. The bite was short, and the man had proved himself capable of breaking anything in the way of porcelain. He had a heavy moustache, and his mouth was already disfigured with anterior restorations in gold, so I had no hesitation in filling the gap with a gold crown attached to crowns on the adjoining teeth. It was simply a hollow dummy open towards the gum, this dummy being filled partially with cement, leaving room, however, for enough pink gutta-percha to make a perfect-fitting saddle resting on the gum. On looking up my records, I found this bridge had been in position eleven years and six months, and the gutta-percha was still soft and the gum on which the piece rested in a perfectly healthy condition. Since then I have used this plan several times with uniformly good results, and in the exceptional cases, where looks do not count and a strong masticating surface is imperative, it answers admirably.

I would like to say one word in regard to Dr. Bogue's paper. To me the *pièce de resistance,* the paper of all others read at that Congress, was the one by Dr. Michaels. Dr. Black has proved to us that all teeth are pretty much the same histologically, and it goes without saying that if we accept this statement we must look for the reason of caries or immunity from caries in the environment of the teeth. Structure being the same practically in all cases, it must be differing surroundings that make different results.

Now, as if in answer to our reasonable question as to what constitutes the environment making for evil and, as a corollary, the environment making for good, Dr. Michaels comes to us with this paper, showing the results of much investigation and many careful analyses of saliva, and in a measure telling us what this evil environment is. Having, then, almost our first knowledge of those conditions of the secretions of the mouth which we have to guard against, we have something definite to work up to in the way of prophylaxis, and it seems to me this is the entering wedge, promising more for the salvation of teeth and scientific prevention than anything we have hitherto had. These researches stand on the same plane of importance with those of Dr. Miller and Drs. Black and Williams.

*Dr. G. A. Mills.*—I do not altogether favor sacrificing sound teeth for bridge-work, though I have a case in my own mouth that justifies it in some instances. I had worn various plates to carry a lost superior central incisor, and for this I substituted a piece of bridge-work. Having a sound lateral incisor, I placed upon it a Richmond crown and attached a dummy central, extending a half-round gold wire collar to the cuspid covering the palatal portion. This I have worn with entire comfort for eighteen years without any repairs.

Dr. Eames has said that local conditions are the beginning of constitutional disorders. Appreciating this remark with what Dr. Cook said at Paris relative to the philosophy of disease, it points to something that will lead to direct intelligence and throw light on such investigations as those of Williams and others in this line of work; also Dr. Michaels's last paper read at Paris, "The Analysis of the Secretions of the Mouth." We predict that these efforts are going to lead us ultimately to valuable conclusions. Dr. Allan has given emphasis to this thought of predisposition and environment, emphasized by Dr. Williams, and we will in the near future have something of fact on which to base a helpful practice. While we cannot destroy the workings of predisposition, we will in no small measure govern it.

*Dr. R. H. M. Dawbarn.*—I was asked a little while ago if I had anything to say with regard to Dr. Brophy's method of operating in cases of congenital cleft palate. I wish to say that I have never yet had a chance to try it. In the three cases which I have had in the past two years where this operation might be considered indicated it has been rejected by the parents. It seems to them, unfortunately, rather severe treatment of a new-born child. Personally I think it a very good operation, and if the patient were my own child I should be inclined to use it. It is not always easy, however, to persuade parents in these cases. I would like to take this occasion to invite you all to my special clinic at the New York Polyclinic Medical School, on Tuesday, January 8. This clinic is to be for dentists especially. The First and Second District Dental Societies of New York and that of Northern New Jersey having asked me to do this at that time, I shall perform an operation by which I shall endeavor to starve a cancer of tonsil and base of tongue and adjacent alveolus by means of cutting off its blood-supply,—that is, by completely excising the external carotid artery and tying off all its branches. It sounds like very heroic treatment, but it has been successful in a number of cases, and in cases where cutting out the growth is impossible it is all we have left which offers hope in such patients. Of course, there is some anastomosis which will still supply sufficient blood to the normal parts for their nourishment; but the growth itself, which requires an abnormally large blood-supply, does not obtain sufficient blood for it to develop further, and it ceases to increase in size, subsequently shrivelling down to a small lump. It has also been suggested, in addition to this ligation, in order to obtain still more radical cutting off of the blood-supply, that the arteries be injected with melted sterilized paraffin, or with very hot water to cause an obliterating endarteritis. This, Dr. Wyeth's proposal, has not as yet been tried. But from the plan of double external carotid excision, now performed by me in over thirty instances (and also at my request by various surgeons of this city and Philadelphia, especially), most encouraging results can be reported, in a few cases the operation now dating back about five years, and without recurrence.

*Dr. Kimball.*—Before adjourning I wish to make a motion of sympathy and thanks to our friend Dr. Bogue, who has, under disadvantageous circumstances, favored us with this paper this evening.

Motion seconded and carried.

*Dr. Howe.*—Gentlemen, I am sure you will all join me in expressing to Dr. Dawbarn our hearty thanks and appreciation for his kind reception of us this evening, and his interesting talk. I think it is an encouragement to all of us to feel that we have a friend whom we can consult, and whose aid we can seek in surgical work outside of our sphere.

Adjourned.

FRED. L. BOGUE, M.D., D.D.S.,
*Editor The New York Institute of Stomatology.*

